# Supplementary material for: An Aqueous Exfoliation of WO3 as a Route for Counterions Fabrication—Improved Photocatalytic and Capacitive Properties of Polyaniline/WO3Composite
Source: Materials (Basel). 2020 Dec 17;13(24):5781. doi: 10.3390/ma13245781 (PMC7766862; doi:10.3390/ma13245781)
Supplement: Supplementary file 1 [file materials-13-05781-s001.zip › materials-1020139-supplementary.pdf]

materials

MDPI

Supplementary Materials

An Aqueous Exfoliation of WO_3 as a Route for Counterions Fabrication—Improved Photocatalyticand Capacitive Properties of Polyaniline/WO_3Composite

Mariusz Szkoda *, Zuzanna Zarach, Konrad Trzciński and Andrzej P. Nowak

Faculty of Chemistry, Department of Chemistry and Technology of Functional Materials, Gdansk University of Technology, Narutowicza 11/12, 80-233 Gdansk, Poland; zuziaz696@gmail.com (Z.Z.); trzcinskikonrad@gmail.com (K.T.); andnowak@pg.edu.pl (A.P.N.)

* Correspondence: mariusz.szkoda1@pg.edu.pl

Received: 14 November 2020; Accepted: 15 December 2020; Published: 17 December 2020

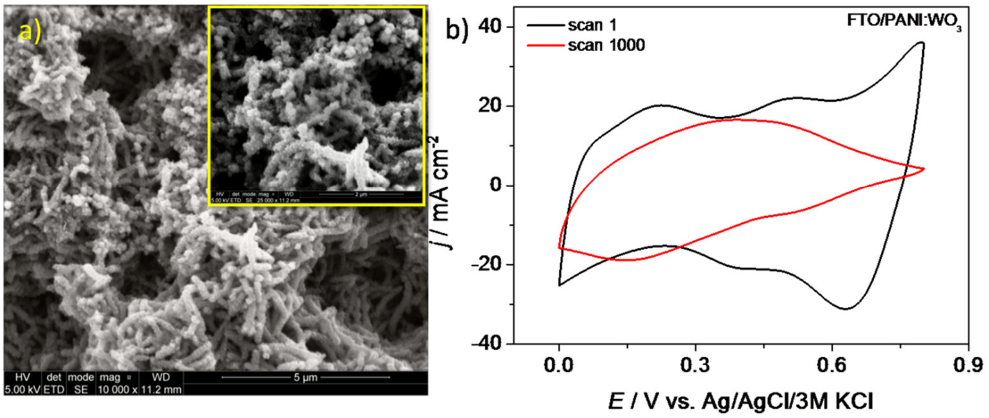

Figure S1. a) SEM images of the FTO/PANI/WO_3 electrode after 1 000 cycles, **b**) Cyclic voltammetry curves (v = 50 mV s^{-1}) before and after 1000 charge and discharge cycles.

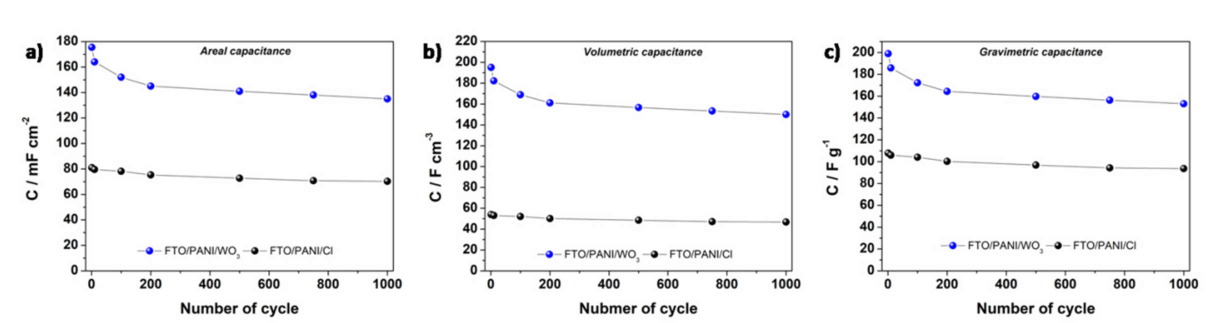

Figure S2. a) The areal capacitance, **b**) the volumetric capacitance and **c**) the gravimetric capacitance vs. number of cycle recorded for a symmetric supercapacitor (FTO/PANI/Cl and FTO/PANI/WO_3).

© 2020 by the authors. Licensee MDPI, Basel, Switzerland. This article is an open access article distributed under the terms and conditions of the Creative Commons Attribution (CC BY) license (http://creativecommons.org/licenses/by/4.0/).
